# Admixture mapping of tuberculosis and pigmentation-related traits in an African–European hybrid cattle population

**DOI:** 10.3389/fgene.2015.00210

**Published:** 2015-06-15

**Authors:** Yonas Kassahun, Valeria Mattiangeli, Gobena Ameni, Elena Hailu, Abraham Aseffa, Douglas B. Young, R. Glyn Hewinson, H. Martin Vordermeier, Daniel G. Bradley

**Affiliations:** ^1^Smurfit Institute of Genetics, Trinity College DublinDublin, Ireland; ^2^Aklilu Lemma Institute of Pathobiology, Addis Ababa UniversityAddis Ababa, Ethiopia; ^3^Armauer Hansen Research InstituteAddis Ababa, Ethiopia; ^4^Centre for Molecular Microbiology and Infection, Imperial College LondonLondon, UK; ^5^TB Research Group, Animal and Plant Health AgencyAddlestone, UK

**Keywords:** *Bos taurus*, *Bos indicus*, SNPs, admixture mapping, *Mycobacterium bovis*

## Abstract

Admixture mapping affords a powerful approach to genetic mapping of complex traits and may be particularly suited to investigation in cattle where many breeds and populations are hybrids of the two divergent ancestral genomes, derived from *Bos taurus* and *Bos indicus.* Here we design a minimal genome wide SNP panel for tracking ancestry in recent hybrids of Holstein–Friesian and local Arsi zebu in a field sample from a region of high bovine tuberculosis (BTB) endemicity in the central Ethiopian highlands. We first demonstrate the utility of this approach by mapping the red coat color phenotype, uncovering a highly significant peak over the *MC1R* gene and a second peak with no previously known candidate gene. Secondly, we exploit the described differential susceptibility to BTB between the ancestral strains to identify a region in which *Bos taurus* ancestry associates, at suggestive significance, with skin test positivity. Interestingly, this association peak contains the toll-like receptor gene cluster on chromosome 6. With this work we have shown the potential of admixture mapping in hybrid domestic animals with divergent ancestral genomes, a recurring condition in domesticated species.

## Introduction

Admixture mapping forms a powerful alternative approach to the ubiquitous genome wide association study design for the discovery of genes that contribute to complex traits. Rather than identifying individual SNP variants that show significant association with phenotype, it seeks to identify segments of chromosome in admixed populations within which ancestry from one parental group diverges from expectation (Smith and O’Brien, 2005).

This approach has several advantages. First, with appropriate trait-population combinations it displays high power. This is manifest most clearly when the ancestral populations to the admixed subjects are divergent in distributions of both genotypes and the targeted phenotypes. Second, although dependent on the generation time-depth since admixture, the SNP density required for a genome wide scan is greatly reduced compared to a typical GWAS experiment, often by over two orders of magnitude. However, a corollary is that genetic mapping resolution is comparatively low. Third, admixture mapping is relatively robust to genetic heterogeneity as a confounder of association (Greenspan et al., 2004; McKeigue, 2005; Winkler et al., 2010).

Although the advantage of admixed subjects for gene detection has been recognized for several decades (Chakraborty and Weiss, 1988), it is only in recent years with high throughput SNP genotyping that the full potential of this design has been realized. The mass human diaspora that proceeded from the age of exploration, e.g., European colonization of the Americas, has resulted in admixed populations of several 100 years establishment that are available for investigation. For example, a genomewide survey of African–American patients with chronic kidney/end stage kidney uncovered convincing evidence that the MHY9 gene is involved in the higher predisposition to the disease conferred by African ancestry (Kopp et al., 2008).

There has been little attention to admixture mapping as an approach in other organisms, despite the potential afforded by the prevalence hybrids of ancestral strains with divergence that exceeds that within humans such as in many domestic animals (Bruford et al., 2003). Domestic cattle offer a particular opportunity. Importantly, there are two major domestic genomes, those of *Bos taurus* and *Bos indicus* that were domesticated from distinct wild populations (Loftus et al., 1994). These genomes are estimated as having diverged 280 Kyr or 56,000 generations ago – about 20 times more generations than calculated in humans since the separation of African and European human ancestors (Murray et al., 2010). This divergence has resulted in marked heritable and important phenotypic differences that should be amenable to gene discovery and also in genomic divergence that facilitates easy genetic identification of chromosome ancestry. Moreover, the two taxa are interfertile and both ancient and recent admixtures are plentiful among world cattle herds, for example within the majority of African livestock (Hanotte et al., 2002). Lastly, cattle are assessed for a range of economically and medically important traits that have importance for both production and potentially assisting in understanding related human biology.

One such trait is bovine tuberculosis (BTB), a chronic respiratory infection caused by *Mycobacterium bovis.* This is an emerging veterinary problem in developing countries and there are several reports that it can also be zoonotic which has serious public health implication (Thoen et al., 2009; Michel et al., 2010; Firdessa et al., 2013; Müller et al., 2013). Ethiopia has the largest cattle herd in Africa, a majority of which is comprised of local zebu breeds but with increasing numbers of imported *Bos taurus* breeds and their hybrids. Susceptibility to BTB has been shown to have a heritable component in European cattle (Brotherstone et al., 2010; Richardson et al., 2014). Also, a comparative analysis of the genetic susceptibility patterns between *Bos taurus* and *Bos indicus* has some suggestion of differential disease risk (Collins-Schramm et al., 2002). Studies in the central highlands of Ethiopia, including regions where BTB is endemic, have shown the prevalence and pathology of the disease significantly skewed toward the *Bos taurus* (Holstein–Friesian) as compared to the local *Bos indicus* (Ethiopian Arsi zebu) cattle (Ameni et al., 2007). Both tuberculosis lesion severity and INF-γ test responses were higher for European *taurus* than zebu cattle (Ameni et al., 2006). BTB related traits have been the subject of both single gene and whole genome association investigations in cattle; SLC11A (NRAMP1) had been previously identified as a susceptibility locus in humans and several bovine studies show significant associations (Bellamy et al., 1998; Barthel et al., 2000; Kadarmideen et al., 2011). Two genome wide SNP array association studies in Holstein–Friesian national herds have given significant results but have, to date, have not been replicated (Finlay et al., 2012; Bermingham et al., 2014).

In this study we develop a low-density genome wide scan for ancestry in cattle and use this to genotype *taurus*-zebu hybrids that are of several generations depth of admixture. Their ancestral populations, Ethiopian Arsi zebu and European Holstein–Friesian, diverge in BTB susceptibility as well as in other phenotypes, including coat color. We first demonstrate the utility of our assay by locating with high significance two loci controlling coat pigmentation differences between these ancestral strains. One of these localization peaks includes the known trait gene MC1R. Secondly, we uncover suggestive evidence for a locus influencing the described divergence in tuberculosis susceptibility between parental strains, as assessed using data from skin testing of herds exposed to known BTB transmission under natural conditions. Interestingly, this peak includes, among other potential causative genes, the toll-like receptor gene cluster on chromosome 6.

The major focus of the project was BTB – the coat color trait was included as an incidental measurement but which nevertheless served as a useful proof of principle of application of the approach in this sample.

## Materials and Methods

### Sampling and DNA Extraction

Crossbred animals (hybrids) from Ethiopian field herds were used for the admixture mapping the owner of each animal was interviewed and the following information were recorded:, age, sex, physical condition score (Nicholson and Butterworth, 1986), pedigree of the animal, whether artificial insemination was used, a photograph on a side view with a study number, and address of the owner. Information was verified with the field veterinarians. Only hybrid animals with a breed history of at least two generations were included in the study. Similarly if artificial insemination was used (where generally the sire is Holstein–Frisian), the animal was removed from the study group, to prevent the inclusion of animals with less than two generations hybrid history.

This work was carried out as part of a large Wellcome Trustproject (see Acknowledgments) investigating TB in field situations in humans and cattle in Ethiopia. The CIDT test results from this larger work have been published in numerous publications, including four referenced here (Ameni et al., 2006, 2007, 2008, 2010). Ethical approval was obtained from the Institutional Review Board (IRB) of ALIPB and from a specially convened committee of Veterinarians.

A total of 10 ml of whole blood was collected from the jugular vein of 585 hybrid Holstein–Friesian/Ethiopian zebus. DNA was extracted from blood using the Archive Pure^TM^ DNA purification kit (5 PRIME GmbH) at the Armauer Hansen Research Institute (AHRI), Addis Ababa, Ethiopia. DNA concentration of each sample was quantified using the fluorescence method, Qubit^TM^. A total of 400–700 ng of DNA was used for analysis.

The breeds used to determine which markers were informative (see SNP Mapping Set) were Ethiopian–Arsizebu (40), Boran (8), Holstein–Friesian from the Bovine Hapmap sample collection plus individuals collected in Ireland (56), Hariana (10), Sahiwal (8), Tharparker (7), plus Bovine Hapmap samples from Brahman (20), and Gir (20).

### Phenotyping of the Hybrid Population (BTB Case/Control and Coat Pigmentation)

The hybrid animals were classified as “reactor” (case) or “non-reactor” (control) to tuberculosis infection using a single comparative intra dermal tuberculin test (CIDT). The test was performed via skin injection of purified protein derivatives (PPDs) which are crude extracts of *M. bovis* (PPD-B) and *Mycobacterium avium* (PPD-A). 0.1 ml of each PPD-B and PPD-A (2500 IU/ml, Animal Health Veterinary Laboratories Agency, Weybridge, UK) were injected into two different sites on the animal neck. The subject was classified as “reactor” (CIDT positive) if the skin thickness at the PPD-B site was higher than at the PPD-A site by at least 4 mm. In order to increase the sensitivity of the test without affecting the specificity, animals with a PPD-B site thickness greater than 2 mm were classified as “possible reactors” and were also included in the “reactor” group for the analysis. This was based on an extensive study in the Selalle region, Ethiopia (Ameni et al., 2008, 2010). The animals were classified as “non-reactors” if the skin thickness at PPD-B site was below 2 mm. Phenotypic coat color scoring for each sample was assigned using photographs taken in the field and using the scale of Hirooka et al. (2002); each animal was assessed for the presence of red coat color and a binary assignment given.

### SNP Mapping Set

Selection of informative markers was carried out by first examining data from the Bovine Hapmap consortium (Gibbs et al., 2009). These markers include a substantial fraction discovered by resequencing a zebu (Brahman) and comparison to the Hereford sequence. SNPs from the Illumina 54001 SNP (Van Tassell et al., 2008) chip data were then added to cover areas of the genome where SNPs from the Hapmap set were missing or did not follow the criteria established. The following criteria were used to choose informative markers from both data sets: absolute allele frequency difference between European *Bos taurus* and *Bos indicus* of 0.6 and above, absolute allele frequency difference between African *Bos taurus* and *Bos indicus* of 0.6 and above and inter marker distance between two consecutive SNPs of on average 3.6 Mb (minimum and maximum distance of 0.1 and 13.9 Mb, respectively, depending on available SNP density) with the aim of even coverage across all autosomes (sex chromosomes were excluded). Furthermore, several additional SNPs from genomic regions of biological importance were added. Genotyping was carried out using the Illumina Golden gate assay and was performed at the Wellcome Trust Centre for Human Genetics, Roosevelt Dr., Oxford, UK.

### Genetic Analyses

SNPs with missing genotype data in the cases and/or controls were removed. Poor quality SNPs and samples were filtered and removed using standard quality thresholds (Greenspan et al., 2004). In order to check the homogeneity of the cases and controls (reactor and non-reactor), EIGENSTRAT (Price et al., 2006) was used to verify that the admixed samples clustered in a position intermediate to the parental and outlying populations. For each individual a local and a genome-wide ancestry, indicated by the proportion of European *Bos taurus*, was estimated using ANCESTRYMAP (Greenspan et al., 2004) using a burn-in period of 100 iterations with 200 follow-on iterations. The stability of the result was monitored by increasing the burn-in period and follow-on iterations by a factor of 10. (1000 burn-in and 2000 follow-on iterations).

ANCESTRYMAP calculates individual ancestry estimates averaged across all individuals to identify genomic regions, where there is enhanced ancestry from one of the parental populations indicating the presence of an ancestry-associated gene nearby. For the binomial admixture scan a prior 30 risk model distribution, from 0.1 to 3.0, was tested for the TB admixture analysis. The overall association was calculated by averaging across all the models (Greenspan et al., 2004). The association between phenotypes and ancestry was quantified based on the outputs of two scores from ANCESTRYMAP. The first is a case-only statistic where a locus-specific score, LOD, is calculated as the log 10 of the ratio between the likelihood of the genotype data at the locus under the risk model and the likelihood of the genotype at the locus assuming that the locus is uncorrelated to the phenotype. A locus-specific LOD score >4 is considered as suggestive significance and > 5 as significant. In line with this calculation, ANCESTRYMAP provides an overall account for association scores by taking the averaged likelihood ratio for associations across all loci in the genome and summarizing evidence of a risk locus anywhere in the genome. In this case, a genome wide score >2 is taken as significant and a value >1 as a suggestive significance score (Greenspan et al., 2004). The second statistic used is the case-control score calculated by comparing the locus specific deviations in European ancestry in cases versus controls at each locus across the genome. The score is taken as a *Z*-score where if there is no phenotype association, the score is expected to normally distribute. The level of locus specific case-control statistical significance is taken as a *Z*-score >3 which correlates to uncorrected nominal *P* < 2 × 10^-3^.

## Results

After quality control measures the final set of genome-spaced markers comprised 662 autosomal SNPs. The proxy parental samples for the admixture under examination were East African zebu Arsi and Boran breeds (48 samples), and European *Bos taurus* Holstein samples (56). The initial set of 585 Holstein–African zebu hybrid genotypes were reduced to 502 by exclusion of 67 samples with an estimated percentage of either East African zebu or European *Bos taurus* ancestry greater than 0.90 and an additional 16 samples due to low genotype call rates, suspected duplication or because they were closely related. The BTB trait admixture analysis was based on a final set of 341 cases and 161 controls and the coat color calculations featured 76 red and 406 not-red individuals.

**FIGURE 1:**
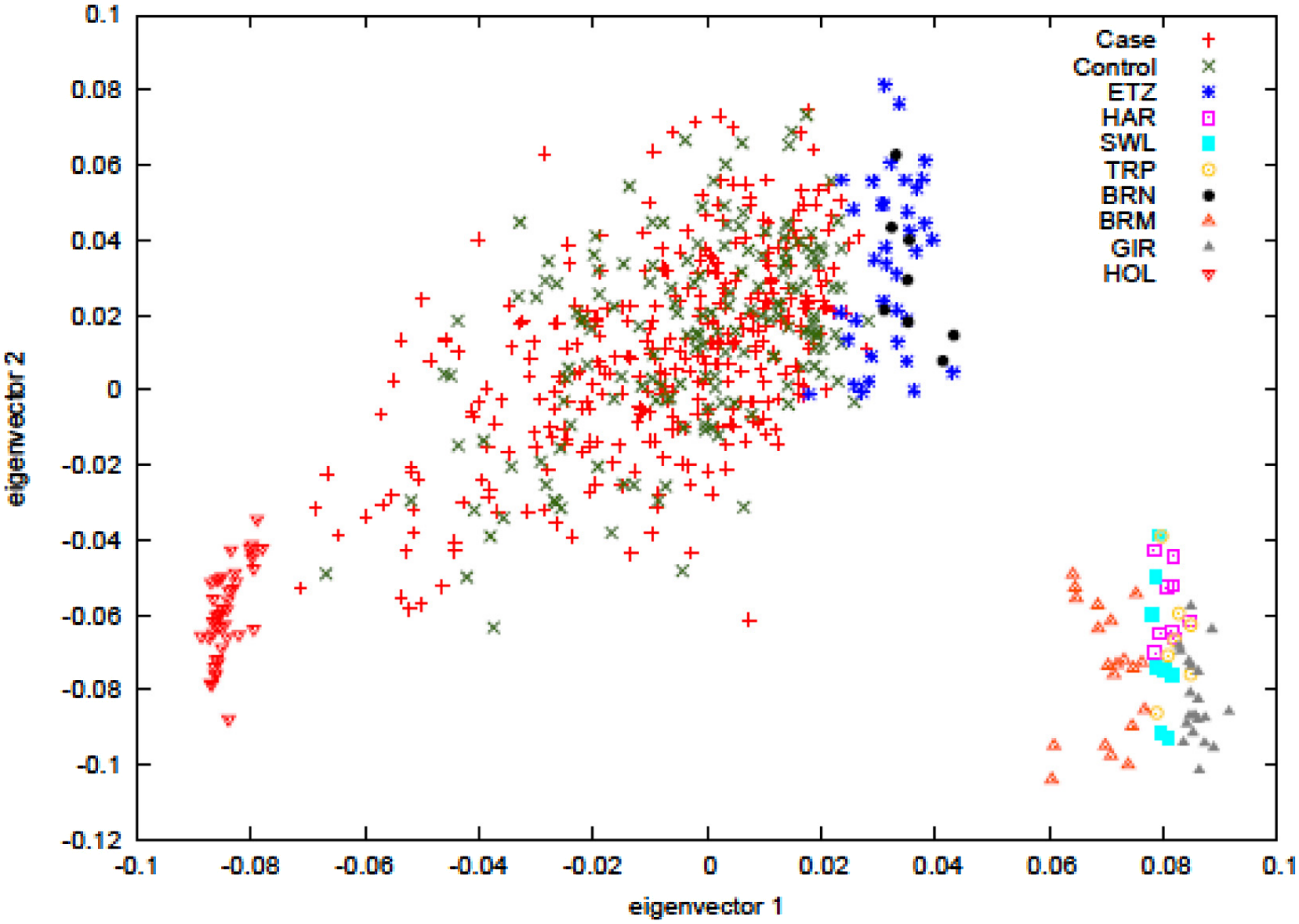
**Principal component analysis of hybrids cases (reactors) and hybrids controls (non-reactors).** The analysis includes the ancestral populations of European *Bos taurus* (Holstein, HOL), East African zebu (Ethiopian zebu – ETZ and Boran – BRN), and non-African zebu (Hariana – HAR, Sahiwal – SWL, Tharparker – TRP, Brahman – BRM, Gir – GIR).

Principal component analysis was carried out to ascertain population variation in relation to the parental populations. The plot in **Figure 1** shows the hybrid case-control animals in the center of the plot while the parental populations, the European *Bos taurus* (HOL) and the East African zebu (ETZ and BRN) cluster at alternate sides of the main distribution, as expected. Non-African zebu separate out with the African zebu on eigenvector 1 but form a clearly distinct cluster. The plot also shows that the hybrid animals have dominant zebu ancestry since most of the individuals are concentrated in close proximity to the zebu parental populations. BTB trait cases and controls are labeled separately and no visible sorting of these was evident.

### Admixture Mapping for Coat Color

A total of 482 hybrids of *Bos taurus* and *Bos indicus*, of a depth of at least two generations, were used in the first admixture analysis using a binary coat color phenotype. For a single trait variable analysis all the samples were divided into two groups: 76 red, which included animals displaying light-red or red coat color and not-red, the 406 remaining animals. The ANCESTRYMAP analysis was performed using the local Ethiopian zebu (Arsi breed) as one of the ancestral populations. This breed has a variable coat color; animals are mostly dark brown but may also be found with a wide spectrum of black, red, and spotted derivate of colors. The second ancestral population is the European *Bos taurus*, Holstein–Friesian. This breed is majority black and white.

**FIGURE 2:**
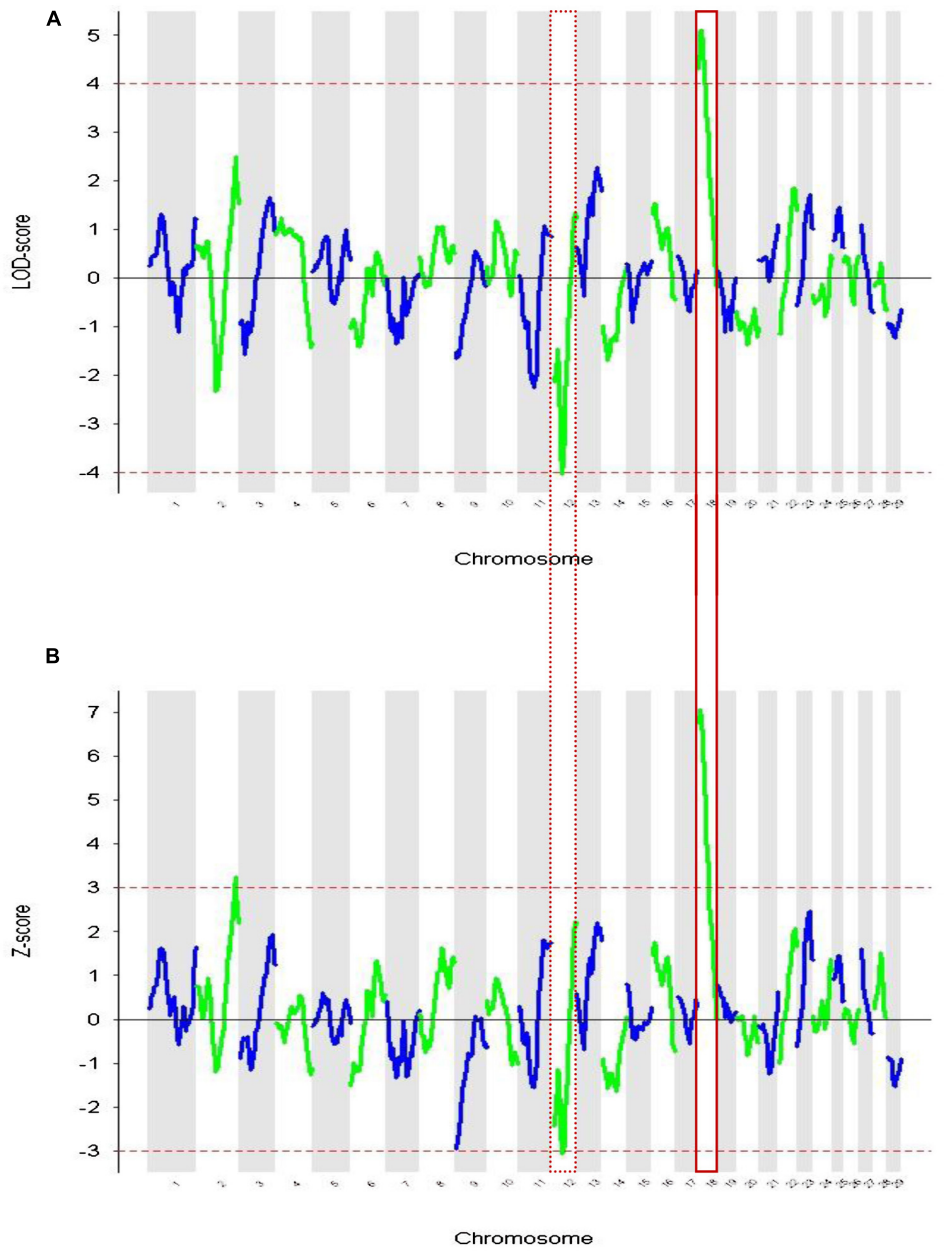
**Admixture mapping of coat color phenotype red vs. not-red for each chromosome. (A)** Showing LOD score based on differences between local ancestry components vs. genomewide levels. **(B)** Plotting the *Z*-scores derived from case-control comparisons. Chromosome 12 and 18 are highlighted because they show significant values in both analyses.

**FIGURE 3:**
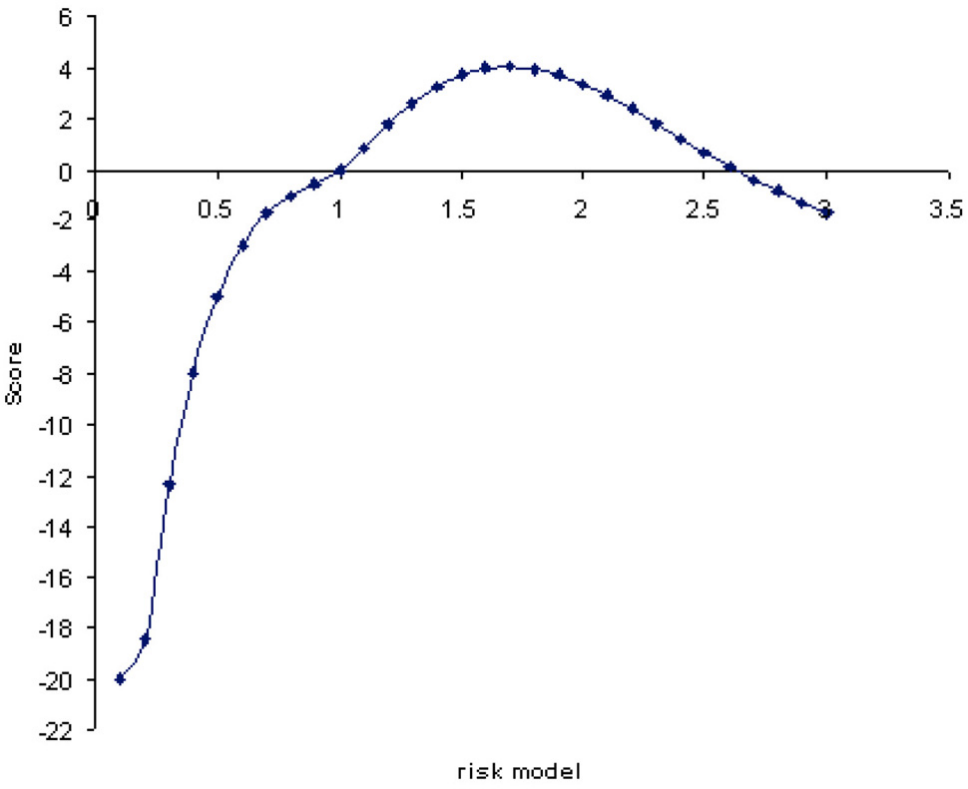
**ANCESTRYMAP risk model (*x*-axis) plotted vs. genomewide average LOD scores.** The maximum likelihood risk model is 1.7 with a genomewide score LOD of value 4.04 (*y*-axis). The average LOD score for all risk models is 3.17.

In **Figure 2** the Z and LOD-scores from the ANCESTRYMAP analysis are plotted. The former is based on a comparison of inferred ancestry levels between cases and controls. The latter is a complementary analysis which assesses locus ancestry levels versus those inferred genomewide for each individual. In both analyses the highest positive score centered in a region of chromosome 18 that contains the melanocortin 1 receptor (*MC1R*) gene (**Figure 3**). This gene is known to regulate the level of tyrosinase enzyme production which is responsible for the switch between phaeomelanin (red pigment) and eumelanin (black pigment) production. The dominant *MC1R* gene has been described as associating with black coat color and a recessive allele results in red coat color (Seo et al., 2007). The *Z*-score was a positive value indicating that the ancestral signal originates within East African zebu. The highest negative score achieved marginal significance (*z*-score = -3.074, LOD = -4.049) in both analyses and was located on chromosome 12. No genes known to be associated with coat color phenotypes were identifiable within this peak region.

### Genomewide Ancestry Association with Tuberculin Positivity

The association between overall genome ancestry and tuberculin reaction was initially tested; **Figure 3** plots the LOD scores associated with risk models ranging from 0.1 to 3-fold increased risk for European *Bos taurus* (Holstein) ancestry. These values are averaged across the genome and correspond to the increase in risk with addition of one copy of a *Bos taurus* ancestry allele. In effect this model tests the association of genomewide European ancestry with tuberculin reaction. The maximum log-likelihood ratio (LOD) was 4.04 for a risk value of 1.7. The average LOD value over each of the risk models considered was 3.17. This overall risk model analysis gives significant evidence of association between European *Bos taurus* ancestry and tuberculin positivity.

### Genome Scans for Association with Tuberculin Positivity

As with red coat color, we implemented two approaches in order to search for genome regions associated with TB susceptibility. First, inferred ancestry at each position was compared in 341 CIDT skin test positives (cases) and 161 test negative controls from the hybrid herds, with 48 East African zebu and 56 European Holstein genotypes used as parental reference samples. Second, inferred ancestry at each position was considered versus a genomewide average for case samples without reference to the control genotypes. These are two non-independent approaches of different power and propensity to false discoveries. We consider only signals that appear with both methods.

**FIGURE 4:**
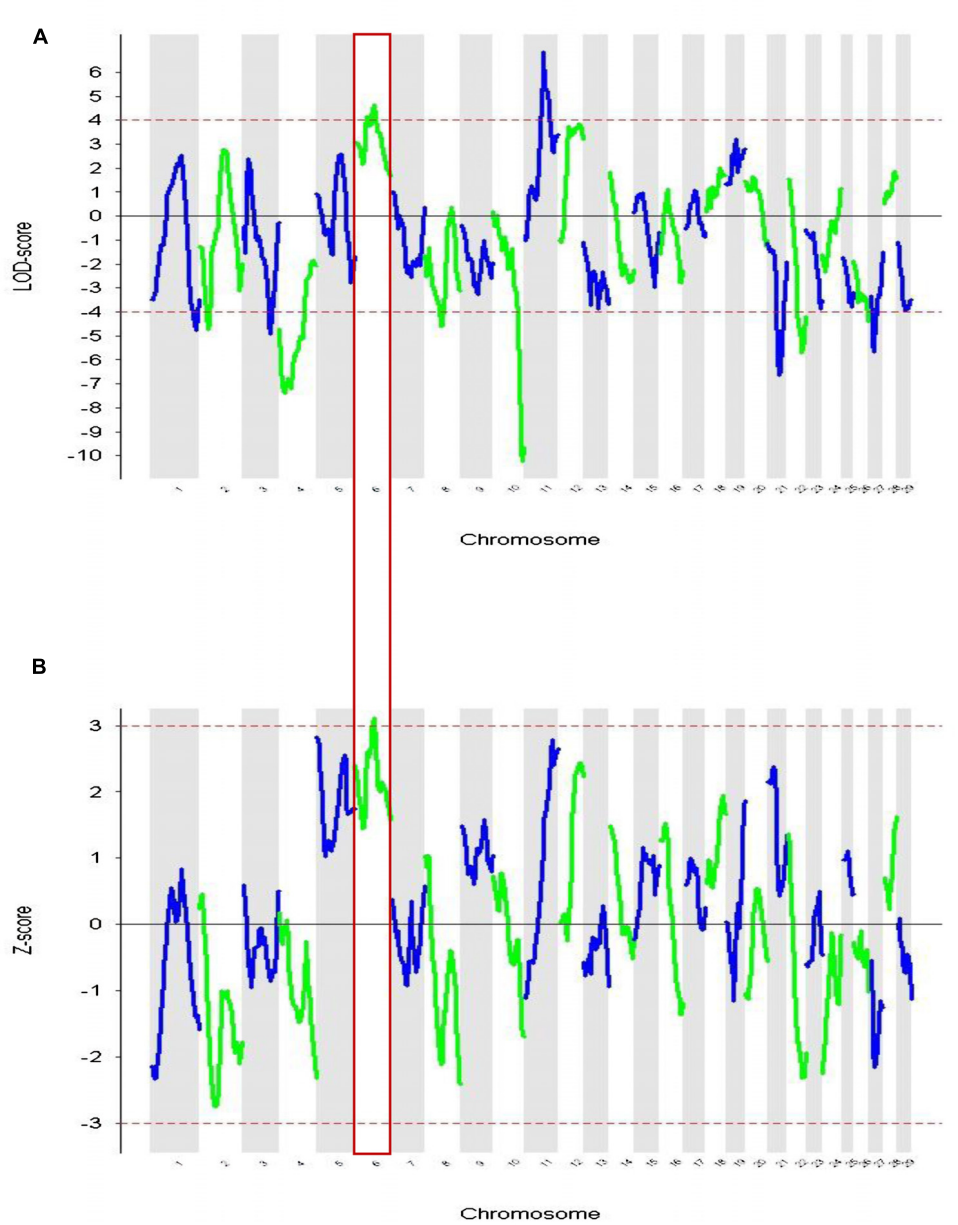
**Admixture mapping of cases vs. controls for each chromosome.** The association of comparative intra dermal tuberculin test (CIDT) positivity with ancestral origin is shown. **(A)** Cases only analysis (LOD) and **(B)** case-control score (*Z*-score). Chromosome 6 is highlighted and has significant values in both analyses.

These admixture mapping scans are plotted for genome position in **Figure 4** with panel A showing LOD scores for the case only analysis and panel B indicating case control comparisons assessed as *Z*-scores. The significance threshold of *Z* = 3.0 and the suggestive significance level of LOD = 4.0 are indicated, respectively. In the case-control comparison, the only significant association found was on chromosome 6 for the marker BTA76573 with a *Z*-score value of 3.11 which corresponds to a nominal *P* = 1.8 × 10^-3^ (**Table 1**).

**Table 1:** SNPs showing the strongest genetic background in the tuberculin positivity association analysis.

Chromosome	Marker	Position (bp)	LOD	*Z*-score	OR (95%CI)	*P*-value
5	BTA149284	2436485	0.94	2.83	0.75 (0.49–1.16)	4.6 × 10^-3^
**6**	**BTA76573**	**65162299**	**4.65**	**3.11**	**1.89 (1.23–2.88)**	**1.8** × **10^-3^**
11	rs29019760	64371895	6.87	1.59	2.59 (1.68–3.99)	1.70 × 10^-5^
12	BTA152656	35296048	3.77	0.12	1.81 (1.18–2.78)	6.79 × 10^-3^
19	BTA133013	35619640	3.23	0.03	1.96 (1.27–3.00)	2.19 × 10^-3^

“Marker “is the SNP identification, “Position” is the position of the SNP on the correspondent chromosome, “OR” is the odd ratio at 95% confidence interval. The marker in chromosome 6 is highlighted, being the only one with both LOD and *Z*-score with significant values.

This locus also has a strong suggestive peak signal for the case only analysis with a LOD score of 4.65. The second highest *Z*-score was on chromosome 5 for the marker BTA149284 (*Z*-score = 2.83; *P* = 4.6 × 10^-3^); however, here the LOD score for case only analysis was not significant (**Table 1**). Within the case only analysis the highest signal observed was for the marker rs29019760 on chromosome 11 (LOD = 6.87), but the value for the case-control comparison analysis was well below significance (*Z*-score = 1.59; **Table 1**). No other LOD score peaks have corresponding case control *Z*-scores with any support for significance.

The region in chromosome 6 associated with the single locus (BTA76573) that showed support for association in each analysis was examined to list possible candidate genes for association with susceptibility. The Bos_taurus_UMD_3.1.1/bosTau8build of the bovine genome data (UCSC genome browser; http://genome.ucsc.edu/) was used to extract genes from the associated region using a one LOD interval (Lander and Botstein, 1989). The list of genes harbored in the candidate region in chromosome 6 is presented in **Table 2**. The region contains the cluster of toll-like receptor genes *TLR1*, *TLR6*, *TLR10*, and the *RHOH* gene. TLRs are important in bacterial pattern recognition and are known to be cellular receptors that play pivotal roles in host innate immune responses (Ryffel et al., 2005; Khor, 2009). RHOH has a role in the development of T cell lymphocytes via modulation of T cell receptor signaling (Wang et al., 2011).

**Table 2:** Genes in the one-LOD interval in chromosome 6 with significant LOD and *Z*-score values.

Chromosome	Marker	Position	Upper–lower boundary	LOD	*Z*-score	Genes
6	BTA76573	65162299	53432843–65162299	4.65	3.11	C6H4orf19, RELL1, PGM2, TBC1D1, KLF3, T**LR10, TLR6, TLR1**, TMEM156, WDR19, KLB, RPL9, LIAS, UGDH, SMIM14, UBE2K, **RHOH**, CHRNA9, RBM47, APBB2, UCHL1, LIMCH1, TMEM33, BEND4, SHISA3, ATP8A1, HTATSF1, KCTD8, YIPF7, GUF1, GNPDA2

“Marker” is the SNP, “Position” is the base pair position of the marker, “Upper–lower boundary” is the upper and lower limits of the gene positions associated with the SNP. The genes highlighted are discussed in the text.

## Discussion

Admixture mapping in humans has had marked success in recent years and displays high power. For example, a sample size of less than 500 was sufficient to effectively scan an African–American population where the differential risk due to ancestry was ∼1.6 (Smith et al., 2004). Such a New world admixture has an average time depth of 10–13 generations and requires ∼2500 markers for a genomewide screen.

Admixture has been a major theme in bovine genetic research (Bradley et al., 1996; Hanotte et al., 2002; Freeman et al., 2006; Berezhnoy et al., 2009; Gibbs et al., 2009), where the two ancestral genomes result from separate domestications of divergent wild populations in the Near East and Indus Valley regions. The genetic distinction between these bovine genomes greatly exceeds any observed between modern human populations and rather, is more akin to that between *Homo sapiens* and archaic *Homo* species (Canavez et al., 2012). Consequently, the availability of markers with strong parental frequency differential is high, facilitating the identification of ancestral origins of genomic regions. Also, genetic divergence is matched by phenotypic difference as different ecologies, domestic breeding and disease challenge histories have led to important trait differences between *Bos indicus* and *Bos taurus* breeds. The former predominate in arid and tropical regions and the latter in temperate climates. The two genomes have several hybrid zones which range in time depth from those potentially at the dawn of herding in West Asia through those from several 1000 years-old migrations of Asian cattle to Africa to recent deliberate crossbreeding for production (Hanotte et al., 2002; Berezhnoy et al., 2009). In recent decades a common practice has been to improve productivity by crossing exotic European high performance stock with local cattle in many regions, including East Africa.

We sought to exploit the population structure of a 3–4 generations deep admixture of European Holstein and local Arsi zebu cattle from the central highland region of Ethiopia where BTB is prevalent. Recently admixed populations have higher levels of admixture generated linkage disequilibrium compared to ancestral populations (Falush et al., 2003; Halder and Shriver, 2003; Hoggart et al., 2004) and this, combined with the efficiencies from ancestral divergences allowed us to design a genomewide admixture mapping screen using only 662 highly informative markers with a high ancestral frequency differential and 502 hybrid test subjects.

As a proof of principle we first mapped the red coat color trait, assessed from visual inspection of subject photographs; the red phenotype is absent from the European parental strain and segregates in the local unimproved cattle. This clearly demonstrated utility of the approach, with a strong significant likelihood peak, confirmed in alternate case-control and case only analyses, over the region with the MC1R locus. This gene has been shown to segregate with coat color and is associated with red pigmentation in a number of mammals, including humans. A second peak on chromosome 12 is less strongly supported result and does not correspond to a known pigmentation locus (Hu et al., 2007). Also, chromosomal resolution is low, of the order of several maga base pairs, as would be expected from a shallow generational history of admixture.

Bovine tuberculosis is an emerging veterinary and public health problem in the developing world. Previous studies reported that crossbred animals in the current study area have intermediate level of tuberculosis prevalence and that European cattle (Holstein) were at least two times more susceptible than the local breeds (Ethiopian zebu; Ameni et al., 2007). Using the same subjects we carried out whole genome admixture mapping to search for genomic regions associated susceptibility in hybrid animals using the CIDT test as a binary trait. This skin test is the standard immunodiagnostic procedure used for the identification of BTB infected cattle. An initial analysis established significant evidence for association between CIDT positive reactions and overall genome levels of *Bos taurus* ancestry. A genomewide scan showed several positions that gave significantly trait-associated levels of one or other parental strain ancestry in either the case only or case-control analyses. However, only one peak was replicated in each that centered on marker BTA76573 on chromosome 6 with a regional excess of Holstein ancestry in cases relative to controls and also relative to the genome average.

Interestingly, this chromosome 6 peak harborsa TLR gene cluster of potential importance to *Mycobacterium* infections. The central roles of TLR1, TLR6, TLR10 in innate immunity are well documented (Heldwein and Fenton, 2002; Reiling et al., 2002; Heldwein et al., 2003; Doherty and Arditi, 2004; Korbel et al., 2008; Möller et al., 2010; Liping et al., 2012). They are part of the Toll gene family that is involved in bacterial pattern recognition (pathogen associated molecular patterns, PAMPs) including BTB and initiate the modulation of an innate immune response (Quesniaux et al., 2004; Möller et al., 2010). Mutations have been implied to confer either resistance or increased susceptibility to infectious diseases in humans (e.g., invasive bacterial infections; Coats et al., 2003), human tuberculosis (Selvaraj et al., 2009; Bryc et al., 2010), and malaria (Corr and O’Neill, 2009) specific to the binding spectrum of the TLRs involved (Khor, 2009). Importantly Sun et al., 2012, in a single gene test also found a significant difference between TLR1 allele frequencies between BTB-infected and non-infected Chinese Holstein cattle cohorts (Sun et al., 2012). However, we caution that the mapped interval in our study is wide – a consequence of the recent admixture of the subjects – and contains multiple other genes that could be considered candidates (**Table 2**).

Prior mapping work for BTB susceptibility in cattle has identified association with the SLC11A1 locus (formerly named NRAMP1) including in African cattle, however, this genome region showed no signal of note in this present study (Barthel et al., 2000; Kadarmideen et al., 2011). Additionally, several genome regions identified as candidates in genome wide association studies of BTB traits in British and Irish herds showed no correspondence here (Finlay et al., 2012; Bermingham et al., 2014). These disjunct results may not be surprising given that the present study is designed to detect variants segregating between zebu and European cattle breeds which are likely different to those that segregate within European herds. Also, scans for tuberculosis susceptibility in humans have identified and replicated fewer loci than for other equivalent genome wide studies of infectious diseases; it may be that genetic effects are made up of many segregating polymorphisms which are each of small contribution due to strong and sustained selective pressure by this widespread pathogen (Curtis et al., 2015).

In summary, we have used a genome wide marker set chosen for high allele frequency divergence between the two genomes segregating in modern cattle typed in a hybrid animal sample to conduct admixture mapping on two traits: susceptibility to tuberculosis and coat color pigmentation. Both approaches give significance peaks that correspond with known candidate loci: MC1R for pigmentation and the TLR cluster on chromosome 6 for BTB susceptibility. This work is a first illustration of the potential of this approach in domestic animals which may have wide and efficient applicability given the prevalence of divergent ancestral genomes and their hybrids in the most important domesticates.

## Conflict of Interest Statement

The authors declare that the research was conducted in the absence of any commercial or financial relationships that could be construed as a potential conflict of interest.
