# Supplementary material for: Admixture mapping of tuberculosis and pigmentation-related traits in an African–European hybrid cattle population
Source: Front Genet. 2015 Jun 15;6:210. doi: 10.3389/fgene.2015.00210 (PMC4467177; doi:10.3389/fgene.2015.00210)
Supplement: Supplementary file 1 [file Table_1.DOCX]

Table S1

Distance between markers in each chromosome

| Chromosome | Average Mb | max Mb | min Mb |
| --- | --- | --- | --- |
| 1 | 4.17 | 10.27 | 1.21 |
| 2 | 3.90 | 11.21 | 0.28 |
| 3 | 3.73 | 9.02 | 0.99 |
| 4 | 4.18 | 9.21 | 0.98 |
| 5 | 3.96 | 10.77 | 1.54 |
| 6 | 4.47 | 9.24 | 1.53 |
| 7 | 3.94 | 8.70 | 0.35 |
| 8 | 3.51 | 8.52 | 0.40 |
| 9 | 3.54 | 7.40 | 0.68 |
| 10 | 3.80 | 7.04 | 0.40 |
| 11 | 3.70 | 7.84 | 1.26 |
| 12 | 3.32 | 9.74 | 0.17 |
| 13 | 3.46 | 7.90 | 0.36 |
| 14 | 3.46 | 6.14 | 1.00 |
| 15 | 4.07 | 13.93 | 0.90 |
| 16 | 3.89 | 10.03 | 1.19 |
| 17 | 3.72 | 9.21 | 1.34 |
| 18 | 3.18 | 6.63 | 0.53 |
| 19 | 2.49 | 5.66 | 0.11 |
| 20 | 3.66 | 9.49 | 0.62 |
| 21 | 3.51 | 7.83 | 1.56 |
| 22 | 3.37 | 5.33 | 0.72 |
| 23 | 3.30 | 5.79 | 0.65 |
| 24 | 3.23 | 7.90 | 0.64 |
| 25 | 3.54 | 5.67 | 2.43 |
| 26 | 3.29 | 5.55 | 1.29 |
| 27 | 3.18 | 5.69 | 0.86 |
| 28 | 3.44 | 2.03 | 3.15 |
| 29 | 2.97 | 5.32 | 1.08 |
