# Supplementary material for: Admixture mapping of tuberculosis and pigmentation-related traits in an African–European hybrid cattle population
Source: Front Genet. 2015 Jun 15;6:210. doi: 10.3389/fgene.2015.00210 (PMC4467177; doi:10.3389/fgene.2015.00210)
Supplement: Supplementary file 2 [file Table_2.DOCX]

Table S2

Markers list

| Chromosome | Position on chromosome (bp) | Marker_ID |
| --- | --- | --- |
| 1 | 2596843 | BTA123586 |
| 1 | 6549125 | BTA123664 |
| 1 | 10312136 | 51416BTA19350 |
| 1 | 12943190 | BTA89190 |
| 1 | 16855656 | BTA118720 |
| 1 | 20434375 | BTA123778 |
| 1 | 24483631 | rs29014870 |
| 1 | 26710108 | rs29016891 |
| 1 | 32619231 | ARSBFGLNGS1179 |
| 1 | 39233890 | BTA115318 |
| 1 | 41201221 | BTA117821 |
| 1 | 45193604 | BTA123915 |
| 1 | 50742649 | BTA152822 |
| 1 | 53907613 | BTB01315615 |
| 1 | 64174818 | BTA153744 |
| 1 | 67443942 | BTA124164 |
| 1 | 70920666 | BTA124117 |
| 1 | 73011709 | BTA124197 |
| 1 | 80204225 | BTA36647 |
| 1 | 84809475 | BTA124292 |
| 1 | 90931560 | BTA155157 |
| 1 | 94466426 | BTA162436 |
| 1 | 99587016 | BTA124441 |
| 1 | 100797998 | rs29010370 |
| 1 | 107487719 | BTA163250 |
| 1 | 110535475 | BTA98374 |
| 1 | 114900005 | BTA124525 |
| 1 | 117791435 | BTA124569 |
| 1 | 120372217 | BTA53011 |
| 1 | 125739110 | BTA22042 |
| 1 | 131319367 | BTA91135 |
| 1 | 135119865 | BFGLNGS115064 |
| 1 | 138657558 | BTA124391 |
| 1 | 145945106 | BTA55339 |
| 1 | 148672481 | BTA19868 |
| 1 | 150948127 | BTA124887 |
| 1 | 156031659 | BTA140446 |
| 1 | 161017702 | BTA33062 |
| 2 | 191747 | BTB01789851 |
| 2 | 4233418 | ss46526186 |
| 2 | 7761986 | BTA47537 |
| 2 | 13662684 | BTA160970 |
| 2 | 18990235 | BTA133627 |
| 2 | 22837188 | BTA46748 |
| 2 | 25351975 | BTA133767 |
| 2 | 28910086 | BTA133796 |
| 2 | 32115274 | BTA157592 |
| 2 | 36674732 | BTA133786 |
| 2 | 40679361 | BTA133922 |
| 2 | 44996947 | BTB01247268 |
| 2 | 49761603 | rs29014403 |
| 2 | 55453507 | BTARHGAP15 |
| 2 | 57279353 | 51787BTA101971 |
| 2 | 61996815 | BTA148285 |
| 2 | 64640561 | BTA47788 |
| 2 | 65744631 | BTA134039 |
| 2 | 72164867 | BTA151467 |
| 2 | 73870231 | BTA134068 |
| 2 | 76284798 | BTA109734 |
| 2 | 87492816 | BTA114127 |
| 2 | 90915354 | BTA111783 |
| 2 | 93963498 | BTA101823 |
| 2 | 99794739 | BTA134315 |
| 2 | 106080269 | BTA154256 |
| 2 | 109524324 | BTA101152 |
| 2 | 112787818 | BTA161118 |
| 2 | 116969661 | BTA134534 |
| 2 | 120238915 | BTA134541 |
| 2 | 124564210 | BTA49215 |
| 2 | 130150578 | BTA134656 |
| 2 | 133564267 | BTA123346 |
| 2 | 136266602 | BTA49846 |
| 2 | 136546910 | BTA41867 |
| 2 | 140621929 | BTA134746 |
| 3 | 5136070 | BTA156199 |
| 3 | 9924889 | SLAM24956 |
| 3 | 10916641 | ARSBFGLNGS40088 |
| 3 | 13934385 | rs29014816 |
| 3 | 17272251 | BTA141008 |
| 3 | 20957596 | BTA141106 |
| 3 | 21973052 | BTA66885 |
| 3 | 28447595 | BTA85203 |
| 3 | 30821637 | BTA141183 |
| 3 | 36297309 | BTA67289 |
| 3 | 39282794 | rs29022833 |
| 3 | 43670979 | BTA20638 |
| 3 | 45148323 | BTA159128 |
| 3 | 50965844 | BTB00462657 |
| 3 | 52012235 | BTA90484 |
| 3 | 56035150 | BTA99527 |
| 3 | 62897338 | BTA141443 |
| 3 | 64244295 | 34680BES106922496 |
| 3 | 66506175 | BTA111096 |
| 3 | 74215124 | BTB00134713 |
| 3 | 78686546 | BTA43649 |
| 3 | 84337697 | BTA102711 |
| 3 | 88185033 | rs29011277 |
| 3 | 90571183 | BTA141572 |
| 3 | 92955808 | BTA95423 |
| 3 | 97087818 | BTA155133 |
| 3 | 102754319 | BTA147456 |
| 3 | 107351079 | BTA103084 |
| 3 | 110717476 | BTA100103 |
| 3 | 119741147 | BTA163309 |
| 3 | 120910004 | BTA89314 |
| 4 | 2262125 | BTA158406 |
| 4 | 7212693 | BTA116504 |
| 4 | 10850192 | BTA155392 |
| 4 | 15383222 | 39584BTA71323 |
| 4 | 16362674 | BTA147009 |
| 4 | 19119397 | rs29021004 |
| 4 | 23819393 | BTA72619 |
| 4 | 28754236 | 50133BTA86017 |
| 4 | 33309069 | BTB00173673 |
| 4 | 39617201 | BTA70234 |
| 4 | 41052720 | BTA155258 |
| 4 | 45766964 | 31113BTA142103 |
| 4 | 51115714 | rs29022883 |
| 4 | 55733521 | BTA152081 |
| 4 | 57045663 | BTA142195 |
| 4 | 62748128 | ARSBFGLNGS56156 |
| 4 | 66290526 | BTA70886 |
| 4 | 69497761 | rs29012383 |
| 4 | 78525246 | BTA71497 |
| 4 | 81641883 | BTA142349 |
| 4 | 84024720 | BTA87133 |
| 4 | 89732026 | BTA25091 |
| 4 | 95706599 | BTA142388 |
| 4 | 99938054 | BTA71764 |
| 4 | 102206584 | BTA19531 |
| 4 | 105873185 | BTA25514 |
| 4 | 115086525 | 33223BTA150780 |
| 4 | 118913503 | BTA142672 |
| 4 | 123600753 | BTA142686 |
| 5 | 2436485 | BTA149284 |
| 5 | 4694787 | ARSBFGLNGS3503 |
| 5 | 8427297 | BTA163543 |
| 5 | 12116513 | ARSBFGLNGS89845 |
| 5 | 16144756 | rs29017027 |
| 5 | 21665722 | BFGLNGS110624 |
| 5 | 26708719 | rs29020008 |
| 5 | 28774242 | BTA72999 |
| 5 | 33793313 | BTA143025 |
| 5 | 37971226 | BTB00572772 |
| 5 | 41154328 | ARSBFGLNGS53488 |
| 5 | 43624692 | BTA27688 |
| 5 | 46025315 | ARSBFGLNGS7850 |
| 5 | 49933340 | BTA143102 |
| 5 | 52454672 | BTA73515 |
| 5 | 54725319 | BTA159936 |
| 5 | 59907178 | BTA143127 |
| 5 | 65689216 | BTA143196 |
| 5 | 71083995 | BTA73745 |
| 5 | 76714910 | ss46526764 |
| 5 | 80876821 | BTA74018 |
| 5 | 84460395 | BTA74086 |
| 5 | 91071209 | BTA74417 |
| 5 | 95977772 | BTA74538 |
| 5 | 99515505 | BTA100895 |
| 5 | 101198827 | BTA30498 |
| 5 | 111966640 | BTA74932 |
| 5 | 114239196 | BTA156468 |
| 5 | 118678294 | BTA164041 |
| 5 | 120216569 | BTA75068 |
| 5 | 125087141 | rs29015304 |
| 6 | 228322 | BTA104879 |
| 6 | 5295340 | BTA101240 |
| 6 | 11042950 | BTA76806 |
| 6 | 14049348 | BTA143762 |
| 6 | 21307414 | BFGLNGS112620 |
| 6 | 27071402 | BTA136088 |
| 6 | 28948477 | BTA157854 |
| 6 | 33903147 | BTC032897 |
| 6 | 36975781 | BTA75979 |
| 6 | 39525430 | BTA144148 |
| 6 | 41379757 | BTC062515 |
| 6 | 46922173 | BTA144031 |
| 6 | 53432843 | rs29026369 |
| 6 | 57806018 | BTA18387 |
| 6 | 65162299 | BTA76573 |
| 6 | 74402801 | BTA144164 |
| 6 | 77304813 | BTA136608 |
| 6 | 85946429 | BTA153462 |
| 6 | 91138928 | BTA77209 |
| 6 | 96979904 | BTA45500 |
| 6 | 102627232 | BTA152644 |
| 6 | 105582191 | BTC046417 |
| 6 | 110790510 | BTA153209 |
| 6 | 112708414 | rs29026829 |
| 6 | 114240706 | BTA90826 |
| 6 | 116601876 | BTA159056 |
| 6 | 120914184 | BTA04482 |
| 7 | 3683688 | BTA79402 |
| 7 | 4033389 | BTA144629 |
| 7 | 4820181 | rs29027201 |
| 7 | 9338124 | ARSBFGLNGS28362 |
| 7 | 12982554 | BTA144755 |
| 7 | 17901772 | TRIF2630 |
| 7 | 21868992 | BTA156653 |
| 7 | 24620081 | BTA144918 |
| 7 | 30070268 | rs29016545 |
| 7 | 34753756 | BTA157608 |
| 7 | 38042437 | BTA150427 |
| 7 | 42141847 | ARSBFGLNGS104510 |
| 7 | 47251264 | BTA150640 |
| 7 | 51136343 | rs29022790 |
| 7 | 52275199 | BTA145091 |
| 7 | 57983805 | BTA79455 |
| 7 | 61118183 | BTA79393 |
| 7 | 65978304 | rs29012520 |
| 7 | 69133028 | rs29018719 |
| 7 | 73129082 | BTA150217 |
| 7 | 80376731 | BTA145282 |
| 7 | 84035438 | BTA151849 |
| 7 | 87171915 | rs29021610 |
| 7 | 95426991 | BTA145358 |
| 7 | 96893151 | 48501BTA87072 |
| 7 | 101287576 | BTA147637 |
| 7 | 109991447 | BTA116247 |
| 8 | 394502 | BTA91856 |
| 8 | 4067310 | BTA159509 |
| 8 | 7131386 | ARSBFGLNGS57214 |
| 8 | 7529282 | BTA145508 |
| 8 | 11693072 | BTA145559 |
| 8 | 14117241 | BTA145599 |
| 8 | 17016654 | rs29010774 |
| 8 | 21137376 | BTA145649 |
| 8 | 24785703 | BTA155636 |
| 8 | 28184892 | ARSBFGLNGS68885 |
| 8 | 29669143 | BTA102634 |
| 8 | 32991845 | BTA20102 |
| 8 | 37296728 | BTA116045 |
| 8 | 41384169 | BTA150345 |
| 8 | 49908415 | BTA143048 |
| 8 | 55026347 | BTA81180 |
| 8 | 57141839 | BTA155431 |
| 8 | 61651546 | BTA154821 |
| 8 | 65994025 | BTA145906 |
| 8 | 66972761 | BTA19344 |
| 8 | 74576637 | BTA148363 |
| 8 | 77818564 | BTA81860 |
| 8 | 83418382 | BTA146037 |
| 8 | 85938408 | BTA146051 |
| 8 | 90470507 | BTA82089 |
| 8 | 95898888 | 38258BTA82187 |
| 8 | 99635999 | rs29025168 |
| 8 | 102524089 | BTA154983 |
| 8 | 105270759 | BTA82575 |
| 8 | 109214273 | BTA82008 |
| 8 | 111454942 | rs29018510 |
| 8 | 114019407 | rs29018748 |
| 8 | 116140121 | BTA24878 |
| 9 | 4316326 | BTA83571 |
| 9 | 8771708 | BTA159047 |
| 9 | 15930978 | BTA146275 |
| 9 | 20971883 | BTA83067 |
| 9 | 26144875 | BTA146355 |
| 9 | 29658951 | DPI30 |
| 9 | 33897036 | BTA146375 |
| 9 | 39368972 | rs29022841 |
| 9 | 46773783 | BTA83620 |
| 9 | 47727167 | BTA100329 |
| 9 | 51438154 | BTA87881 |
| 9 | 52952158 | BTA17293 |
| 9 | 57595190 | BTA104642 |
| 9 | 59305310 | rs29023173 |
| 9 | 60819485 | BTA70575 |
| 9 | 64776718 | BTA83985 |
| 9 | 68799465 | BTA157666 |
| 9 | 71435003 | BTA146623 |
| 9 | 72111835 | BTA97753 |
| 9 | 78545481 | BTA160540 |
| 9 | 84194715 | BTA146732 |
| 9 | 90016767 | BTA80583 |
| 9 | 92122143 | 40028BTA84686 |
| 9 | 94666840 | BTA146842 |
| 9 | 96229189 | BTA84872 |
| 9 | 102345316 | BTA153170 |
| 9 | 103899330 | ARSBFGLNGS4320 |
| 9 | 105350884 | BTA146939 |
| 9 | 107022980 | BTA154830 |
| 10 | 3208775 | BTA149082 |
| 10 | 8028208 | BTA125086 |
| 10 | 8429875 | rs29016948 |
| 10 | 9058148 | BTA27577 |
| 10 | 15034937 | rs29023635 |
| 10 | 17177811 | ARSBFGLNGS30236 |
| 10 | 21194044 | rs29020989 |
| 10 | 25244764 | BTA125260 |
| 10 | 28629902 | BTA62307 |
| 10 | 31589546 | BTA133067 |
| 10 | 33261299 | BTA64301 |
| 10 | 36581064 | BTA125429 |
| 10 | 42530888 | BTA117967 |
| 10 | 46458927 | BTA150419 |
| 10 | 51706576 | BTA70219 |
| 10 | 53553739 | BTA125606 |
| 10 | 60545672 | BTA66847 |
| 10 | 63536820 | BTA72369 |
| 10 | 67252021 | BTA153983 |
| 10 | 72770200 | BTA88280 |
| 10 | 78749663 | BTA75328 |
| 10 | 83862921 | BTA29800 |
| 10 | 90901712 | BTA125851 |
| 10 | 94038231 | BTA162780 |
| 10 | 97500100 | BTB01147175 |
| 10 | 100272735 | BTA111054 |
| 10 | 105858246 | BTA151025 |
| 11 | 2568606 | BTA126066 |
| 11 | 4684784 | BTA09207 |
| 11 | 7681930 | BTA101086 |
| 11 | 12827954 | ARSBFGLNGS25668 |
| 11 | 14740201 | ARSBFGLNGS40117 |
| 11 | 15999935 | BTA58740 |
| 11 | 21929482 | 42710BTA86602 |
| 11 | 23709515 | BTA151182 |
| 11 | 27453830 | BTB01550704 |
| 11 | 29051435 | BTA163042 |
| 11 | 31820330 | 27192BTA152811 |
| 11 | 35475727 | BTA92801 |
| 11 | 39253555 | rs29021599 |
| 11 | 40659560 | BTA119892 |
| 11 | 46076502 | BTA126465 |
| 11 | 51159646 | BTA97093 |
| 11 | 56910615 | 42420BTA109576 |
| 11 | 60549611 | BTA154095 |
| 11 | 64371895 | rs29019760 |
| 11 | 69066925 | BTA126653 |
| 11 | 74737884 | BTA126740 |
| 11 | 81528883 | BTA126799 |
| 11 | 89018683 | BTA109346 |
| 11 | 90580366 | BTA111323 |
| 11 | 92719286 | ARSBFGLNGS17353 |
| 11 | 94630901 | BTA149792 |
| 11 | 97395635 | ARSBFGLNGS83728 |
| 11 | 102121773 | ss46526514 |
| 11 | 109959239 | BTA127208 |
| 12 | 8677831 | BTB01099458 |
| 12 | 8844711 | rs29016194 |
| 12 | 9713260 | rs29018847 |
| 12 | 19455973 | BTA127389 |
| 12 | 22970081 | BTA127428 |
| 12 | 27607460 | BTA142670 |
| 12 | 31593883 | BTA127469 |
| 12 | 32537680 | BTA127519 |
| 12 | 35296048 | BTA152656 |
| 12 | 38883979 | BTA102656 |
| 12 | 43406630 | rs29011179 |
| 12 | 47486001 | BTA127628 |
| 12 | 48122821 | BTA50109 |
| 12 | 50938596 | BTA88515 |
| 12 | 54966075 | BTA127704 |
| 12 | 56672506 | BTA19412 |
| 12 | 62069643 | BTB01537669 |
| 12 | 65188345 | BTA162624 |
| 12 | 67648544 | BTA153928 |
| 12 | 71559508 | BTA127797 |
| 12 | 74749956 | BTA149079 |
| 12 | 78783490 | rs29016058 |
| 12 | 85085474 | BTA127953 |
| 13 | 1282960 | BTA123519 |
| 13 | 5480565 | rs29027604 |
| 13 | 13381787 | BTA128109 |
| 13 | 17348597 | BTA128131 |
| 13 | 21950590 | BTA147047 |
| 13 | 24765905 | BTA128187 |
| 13 | 28925713 | rs29012117 |
| 13 | 34570608 | BTA32324 |
| 13 | 37587223 | BTA157942 |
| 13 | 42898449 | BTA32705 |
| 13 | 46918318 | rs29026913 |
| 13 | 48844067 | BTA154675 |
| 13 | 51473531 | BTA98332 |
| 13 | 55014300 | BTA159623 |
| 13 | 56944692 | BTA33033 |
| 13 | 60947573 | BTA160398 |
| 13 | 67687693 | BTA104547 |
| 13 | 69642461 | BTA33460 |
| 13 | 71985168 | ARSBFGLNGS71175 |
| 13 | 72347296 | ARSBFGLNGS23438 |
| 13 | 75409752 | 57019rs29020756 |
| 13 | 78189197 | BTA128752 |
| 13 | 81782108 | BTA34053 |
| 13 | 84221051 | BTA147737 |
| 14 | 1001775 | BTC002234 |
| 14 | 4736993 | ARSBFGLBAC10172 |
| 14 | 7529761 | BTA128920 |
| 14 | 11099144 | BTA156915 |
| 14 | 15833033 | BTA128979 |
| 14 | 16832151 | BTA36014 |
| 14 | 21668492 | BTA34364 |
| 14 | 23519449 | BTA34282 |
| 14 | 25973401 | BTA28253 |
| 14 | 28378897 | BTA34429 |
| 14 | 31184876 | BTA129087 |
| 14 | 35440811 | BTA151811 |
| 14 | 40552301 | BTA158016 |
| 14 | 46190654 | BTA113386 |
| 14 | 50427099 | BTA153618 |
| 14 | 52567916 | BTC061592 |
| 14 | 58709447 | BTA35201 |
| 14 | 61618241 | BTA90526 |
| 14 | 65478823 | BTA129366 |
| 14 | 71228051 | 50329BTA35492 |
| 14 | 73977952 | UAIFASA8066 |
| 14 | 75516539 | BTA129517 |
| 14 | 80537023 | BTA35797 |
| 15 | 2675525 | BTA67640 |
| 15 | 4036305 | ARSBFGLNGS102429 |
| 15 | 12129706 | BTA37442 |
| 15 | 18646517 | rs29019566 |
| 15 | 19543711 | BTA164317 |
| 15 | 21921048 | BTA153449 |
| 15 | 24287936 | BTA96059 |
| 15 | 29436729 | BTA129737 |
| 15 | 33725783 | 45486BTA36522 |
| 15 | 37542589 | BTA161073 |
| 15 | 41084554 | BTA36820 |
| 15 | 55011002 | BTA150498 |
| 15 | 60037017 | ARSBFGLNGS108364 |
| 15 | 63663324 | BTA37250 |
| 15 | 67148006 | BTA147952 |
| 15 | 68253133 | rs29009903 |
| 15 | 69820854 | BTA130177 |
| 15 | 77693328 | BTA130272 |
| 15 | 81414654 | BTA130289 |
| 15 | 84155278 | 24864BTA155172 |
| 16 | 3462171 | BTA130386 |
| 16 | 4656758 | BTB01199899 |
| 16 | 10720854 | BTA39884 |
| 16 | 12395850 | BTA117814 |
| 16 | 15507710 | BTA86900 |
| 16 | 19527696 | BTA150849 |
| 16 | 23320958 | rs29026035 |
| 16 | 27760481 | BTA148687 |
| 16 | 31049009 | BTA108300 |
| 16 | 35978574 | BTA100038 |
| 16 | 38564925 | BTA130637 |
| 16 | 48596192 | BTA130962 |
| 16 | 53743308 | BTA39603 |
| 16 | 58325876 | BTA39712 |
| 16 | 63283574 | rs29013788 |
| 16 | 65996953 | BTA159004 |
| 16 | 69517440 | BTA131223 |
| 16 | 72484382 | BTA104703 |
| 16 | 77378145 | BTA40214 |
| 17 | 11293757 | rs29013191 |
| 17 | 17949289 | SCAFFOLD24042552453 |
| 17 | 20028564 | BFGLNGS118918 |
| 17 | 24689011 | BTA40646 |
| 17 | 33903237 | BTA135174 |
| 17 | 36593831 | IL26031 |
| 17 | 41375661 | rs29025650 |
| 17 | 44010948 | BTA153912 |
| 17 | 47868434 | BTA131422 |
| 17 | 50678168 | BTA123144 |
| 17 | 56519964 | BTA41156 |
| 17 | 57890891 | ARSBFGLNGS42534 |
| 17 | 63033323 | rs29012758 |
| 17 | 67279324 | BTA131630 |
| 17 | 68620699 | BTA150604 |
| 17 | 72755069 | rs29013344 |
| 17 | 74610587 | ss46527108 |
| 18 | 1822486 | BTA131863 |
| 18 | 7393707 | BTA44340 |
| 18 | 7926194 | BTA91698 |
| 18 | 11002597 | BTA44427 |
| 18 | 13030708 | BTA132064 |
| 18 | 18072666 | BTA132100 |
| 18 | 23383717 | BTA42917 |
| 18 | 24474537 | ss46526963 |
| 18 | 27231276 | BTA162019 |
| 18 | 30585398 | BTA162049 |
| 18 | 35985225 | BTA43060 |
| 18 | 38535577 | BTA90173 |
| 18 | 43455975 | BTA132334 |
| 18 | 46881880 | BTA18099 |
| 18 | 51078088 | ss46526490 |
| 18 | 52324967 | BTA43478 |
| 18 | 57282795 | BTA43920 |
| 18 | 63913290 | BTA88433 |
| 18 | 64727406 | ARSBFGLNGS9848 |
| 18 | 65485958 | BTA132631 |
| 19 | 3291696 | BTA102444 |
| 19 | 4483869 | BTA46428 |
| 19 | 5395508 | ARSBFGLNGS3016 |
| 19 | 9616046 | BTA132760 |
| 19 | 12293536 | ARSBFGLNGS26874 |
| 19 | 13003586 | BTA97844 |
| 19 | 16653222 | BTA44565 |
| 19 | 19938235 | BES106051154 |
| 19 | 22921250 | BTA44795 |
| 19 | 25598691 | BTA155051 |
| 19 | 28452400 | BTA114086 |
| 19 | 32317789 | 59534rs29022537 |
| 19 | 32865864 | BTA122803 |
| 19 | 35619640 | BTA133013 |
| 19 | 39709510 | BTA45352 |
| 19 | 41718215 | BTA133166 |
| 19 | 45901285 | ARSBFGLNGS24479 |
| 19 | 46014590 | BTA133186 |
| 19 | 47869682 | UAIFASA2616 |
| 19 | 53525831 | rs29015135 |
| 19 | 56560923 | SCAFFOLD1303511739 |
| 19 | 60083629 | BTA133415 |
| 19 | 61584414 | 52490rs29011071 |
| 19 | 63018297 | 42222BTA46363 |
| 20 | 2256104 | ARSBFGLNGS74477 |
| 20 | 3969362 | ARSBFGLNGS87772 |
| 20 | 5352138 | BTA163364 |
| 20 | 10096641 | BTA122946 |
| 20 | 10897222 | ARSBFGLNGS102102 |
| 20 | 11662775 | ARSBFGLNGS22729 |
| 20 | 14275030 | BTA134915 |
| 20 | 18056193 | BTA136989 |
| 20 | 21019644 | BTA49985 |
| 20 | 26966402 | BTA50080 |
| 20 | 29110847 | 43595BTA50145 |
| 20 | 34367586 | BTA50231 |
| 20 | 38899528 | BTA50468 |
| 20 | 39518858 | BTA13793rs29018751 |
| 20 | 46453651 | BTA55581 |
| 20 | 54753835 | ARSBFGLNGS79994 |
| 20 | 59583788 | BTA85612 |
| 20 | 60208102 | BTA50852 |
| 20 | 69699955 | BTA135353 |
| 20 | 73543290 | BTA117330 |
| 21 | 3985756 | BTA135547 |
| 21 | 11814707 | BTA135646 |
| 21 | 15687206 | BTA123062 |
| 21 | 18090676 | BTA135712 |
| 21 | 23448892 | BTA158721 |
| 21 | 25155413 | rs29022043 |
| 21 | 29098079 | rs29012450 |
| 21 | 33187526 | ARSBFGLNGS12511 |
| 21 | 35149769 | BTA135901 |
| 21 | 38169536 | BTA52183 |
| 21 | 40965382 | BTA135951 |
| 21 | 46285436 | BTA135970 |
| 21 | 47846194 | BTA136015 |
| 21 | 50527043 | rs29012295 |
| 21 | 55762339 | BTA52526 |
| 21 | 58738055 | BTA52652 |
| 21 | 63635184 | ARSBFGLNGS109009 |
| 21 | 67177207 | BTA53093 |
| 22 | 2942820 | BTA136233 |
| 22 | 6998996 | BTA136332 |
| 22 | 9021664 | BTA55165 |
| 22 | 14211803 | ARSBFGLNGS42352 |
| 22 | 16546944 | BTA136356 |
| 22 | 21875220 | BTA53851 |
| 22 | 25810272 | BTA53929 |
| 22 | 30999911 | BTA136529 |
| 22 | 34739911 | BTA157851 |
| 22 | 37193843 | BTA155747 |
| 22 | 42080836 | BTA162186 |
| 22 | 46268111 | BTA54620 |
| 22 | 48919699 | BTA162910 |
| 22 | 53649797 | BTA23509 |
| 22 | 56128327 | BTA100290 |
| 22 | 56845960 | BTA136760 |
| 22 | 60180896 | BTA136812 |
| 23 | 3805227 | BTA56589 |
| 23 | 7957639 | BTA57114 |
| 23 | 13656928 | BTA161953 |
| 23 | 19448374 | BTA158271 |
| 23 | 24975294 | rs29023210 |
| 23 | 27895932 | BTA121396 |
| 23 | 32330068 | BTA56291 |
| 23 | 36629047 | ARSBFGLNGS31956 |
| 23 | 37276657 | BTA154401 |
| 23 | 40251495 | rs29027406 |
| 23 | 43447673 | BTA35669 |
| 23 | 45451358 | BTA56712 |
| 23 | 50745314 | BTA163164 |
| 23 | 51820174 | BTA137529 |
| 23 | 53253289 | BTA152213 |
| 24 | 317204 | BTA154117 |
| 24 | 8215551 | rs29025484 |
| 24 | 10808268 | BTA28187 |
| 24 | 14311904 | BTA158465 |
| 24 | 20966645 | BTA157791 |
| 24 | 22207910 | rs29013920 |
| 24 | 24737826 | BTA137689 |
| 24 | 29609631 | BTA155749 |
| 24 | 31175220 | BTA161589 |
| 24 | 35614236 | BTA137746 |
| 24 | 38922798 | BTA137772 |
| 24 | 40905577 | BTA152408 |
| 24 | 45090507 | BTA58205 |
| 24 | 47665750 | BTA89210 |
| 24 | 51201344 | BTA58574 |
| 24 | 55711846 | BTA160255 |
| 24 | 56354733 | BTA58434 |
| 24 | 58833844 | BTA138041 |
| 24 | 62744174 | BTA114768 |
| 24 | 64946690 | BTA105335 |
| 25 | 5445064 | BTA94060 |
| 25 | 10141656 | BTA60712 |
| 25 | 14247522 | BTA138325 |
| 25 | 17547528 | BTA59167 |
| 25 | 21424196 | ARSBFGLNGS103867 |
| 25 | 25088004 | BTA98894 |
| 25 | 27513325 | BTA110447 |
| 25 | 29965631 | BTA59807 |
| 25 | 35638645 | ARSBFGLNGS1323 |
| 25 | 40854872 | rs29021297 |
| 26 | 627739 | BTA138668 |
| 26 | 5767915 | BTA152874 |
| 26 | 8734787 | BTA113604 |
| 26 | 12188146 | BTA62051 |
| 26 | 16080541 | SCAFFOLD373916456 |
| 26 | 19831953 | BTA138776 |
| 26 | 23510130 | BTA60882 |
| 26 | 25707395 | BTA138880 |
| 26 | 31254032 | BTA138892 |
| 26 | 33869282 | BTA147594 |
| 26 | 38419991 | BES5509942 |
| 26 | 42127791 | BTA61648 |
| 26 | 46838420 | rs29009912 |
| 26 | 48123884 | BTA139158 |
| 26 | 50027686 | SCAFFOLD10503627060 |
| 27 | 5827480 | BTA157233 |
| 27 | 10504491 | 24342BTA147970 |
| 27 | 14415472 | BTA63256 |
| 27 | 16397839 | BTA62194 |
| 27 | 18490749 | 25499BTA123322 |
| 27 | 22645173 | BTA160871 |
| 27 | 27059870 | ARSBFGLNGS67277 |
| 27 | 31098801 | BTA97726 |
| 27 | 35694230 | BTA139459 |
| 27 | 37382759 | BTA158280 |
| 27 | 43073711 | BTA139521 |
| 27 | 43929941 | ARSBFGLNGS62589 |
| 27 | 47158845 | BTA63034 |
| 28 | 2877186 | BTA100910 |
| 28 | 6031352 | ARSBFGLNGS27677 |
| 28 | 9309063 | ARSBFGLNGS112517 |
| 28 | 13997764 | ARSBFGLNGS62227 |
| 28 | 19839375 | BTA92767 |
| 28 | 21480907 | BTA139892 |
| 28 | 26353940 | BTA139962 |
| 28 | 29365953 | BTA140048 |
| 28 | 34265756 | BTA131584 |
| 28 | 39827706 | BTA140153 |
| 28 | 42128901 | BTA140176 |
| 28 | 44154802 | BTA140185 |
| 29 | 2198436 | BTA160713 |
| 29 | 6703584 | BTA109864 |
| 29 | 9819145 | BTA140385 |
| 29 | 12196092 | rs29015214 |
| 29 | 17109723 | rs29022136 |
| 29 | 18185585 | BTA140491 |
| 29 | 21075100 | BTA161100 |
| 29 | 24654997 | BTA161784 |
| 29 | 27045527 | BTA140534 |
| 29 | 28265179 | BTA65181 |
| 29 | 30305655 | ARSBFGLNGS100024 |
| 29 | 34071530 | BTA140580 |
| 29 | 37890596 | BTA142988 |
| 29 | 41431277 | BTA140682 |
| 29 | 46748615 | rs29025626 |
